# Channel estimation based on superimposed pilot and weighted averaging

**DOI:** 10.1038/s41598-022-14482-6

**Published:** 2022-06-18

**Authors:** Qun Wu, Xiao Zhou, Chengyou Wang, Zhiliang Qin

**Affiliations:** 1 School of Mechanical, Electrical and Information Engineering, Shandong University, Weihai, 264209 China; 2 Weihai Beiyang Electric Group Co. Ltd., Weihai, 264209 China

**Keywords:** Engineering, Electrical and electronic engineering

## Abstract

Channel estimation based on superimposed pilot (SP) is a challenge in orthogonal frequency division multiplexing (OFDM) systems. To reduce the pilot data interference in the SP and estimate the channel state information accurately, a weighted averaging (WA) channel estimation method based on the superimposed pilot is proposed in this paper. At the transmitter, two signals with data symbols and pilot symbols superimposed at different subcarriers are transmitted. At the receiver, the elimination scheme is proposed to remove the pilot data interference. Based on the temporal correlation of wireless channels, the WA method is used to reduce the interference caused by additive white Gaussian noise in the channel. Simulation results verify that the proposed method can be applied to different channel scenarios. It has better normalized mean square error and bit error rate performance than other existing methods, and the superimposed pilot can improve the throughput of wireless communication systems.

## Introduction

Orthogonal frequency division multiplexing (OFDM) technology, as one of the key technologies of the 5th generation (5G) mobile communication, has been widely used in modern wireless communication systems due to its higher spectrum efficiency and robustness to wireless channel frequency selectivity^1^. Interference in the channel will cause the transmitted signal to fade. To recover the signal accurately at the receiver, channel estimation is essential^2^. Channel estimation methods for OFDM systems have been exhaustively studied, which can generally be divided into three categories: blind channel estimation^3^, semi-blind channel estimation^4^, and pilot-based channel estimation^5^. Compared with pilot-based channel estimation, blind and semi-blind channel estimation methods have higher spectrum efficiency but are limited by higher computational complexity. Therefore, pilot-based channel estimation methods are more popular in practical applications. Abdzadeh et al.^6^ employed the basis expansion modeling (BEM) to represent the time-varying channel. The Schmidt-extended Kalman filtering^7^ and particle filtering^8^ are used to estimate the carrier frequency offset and BEM coefficients. Gong et al.^9^ considered the time-varying characteristics of different symbols and adopted a new interpolation method to introduce generalized spatial modulation-OFDM technology into the high-speed railway wireless communication system. In pilot-based channel estimation, it can be divided into comb pilot^10^, block pilot^9^, and scattered pilot^11^ according to the way of pilot insertion. Although the channel can be easily estimated by inserting pilot sequences into the transmitted data, it will lead to undesirable loss of data-rate and reduce spectrum efficiency^12^.

Superimposed pilot (SP)^13^ is based on other types of pilot^9–11^, and the data symbols are superimposed with pilot symbols, which can avoid the reduction of spectral efficiency and data rate. Moreover, compared with the conventional pilot scheme, it increases the available bandwidth^14^. The SP scheme is very attractive in most wireless communication systems, especially in 5G communication systems with high data rate requirements^15^. 5G technologies use SP to overcome existing intractable issues, such as the pilot contamination in massive multiple input multiple output (MIMO) systems^16–18^, or combined with new technologies like nonorthogonal multiple access^19^. Lago et al.^16^ proposed a pilot decontamination method in time division duplex massive MIMO systems. The method combines SP with the block pilot to estimate channel state information (CSI) prior to downlink data transmission, and subsequently the CSI obtained by the block pilot is used to reduce the interference caused by transmitting pilot and data together. Zhang et al.^17^ studied the cell-free massive MIMO system with SP for the first time and derived closed-form linear minimum mean square error (LMMSE) channel estimation. Zhang et al.^18^ mathematically characterized the effect on the performance of massive MIMO systems. They concluded that even if the number of base station antennas tends to infinity, the pilot contamination and the pilot data interference (PDI) caused by SP will not vanish.

The PDI will make channel estimation difficult. Conventional pilot-assisted channel estimation algorithms, such as least square (LS)^20^, minimum mean square error^21^, and LMMSE^22^, are not effective in SP-based channel estimation. There is much literature on SP-based channel estimation^15,23–27^. Estrada et al.^15^ proposed a superimposed model on a pre-coded data scheme, which introduces an interference control factor to determine the proportion of pilot power in the SP. They also proposed a data detection method to improve the bit error rate (BER) performance. On the basis of^15^, Zhang and Sheng^23^ moved the superposition index to the position where the power sum of data and pilot is minimum, which further improves the accuracy of channel estimation. Bao et al.^24^ proposed a method that transmits superimposed pilots before precoding the data and pilot separately. Only one waveform is needed to realize the two functions of channel estimation and data detection at the receiver. However, the channel estimation accuracy will decrease as the Doppler shift increases. Gong et al.^25^ proposed an SP-based two-phase channel estimation scheme for the unmanned aerial vehicle assisted cellular communication system, and the scheme considers various unitarily-invariant channel statistics errors. Estrada et al.^26^ proposed a superimposed training approach for channel estimation in multiple-input single-output direct current biased OFDM visible light communication scenarios. Simultaneously, the analytical expressions of mean square error (MSE) and spectral efficiency are derived when the LS estimator is considered. Liao et al.^27^ proposed an iterative extended Kalman filter channel estimation method based on the BEM. This method can be used in high-speed mobile scenarios while the computational complexity of the algorithm is relatively high. SP can also be used for channel estimation of underwater acoustic communication. Yang et al.^28^ developed a message-passing-based bidirectional channel estimation method for underwater acoustic communication and combined the method with SP to obtain accurate CSI and mitigate inter-symbol interference (ISI). However, the method can only transmit the data that follows the Gaussian distribution, which limits its scope of application. Yang* et al*.^29^ proposed a time-varying underwater acoustic channel estimation and equalization method based on SP and low-complexity Turbo equalization in the frequency domain. However, the method requires multiple iterations and is computationally complex.

Because of the temporal correlation of wireless channels, multi-frame averaging (FA) can be used to estimate the channels^30–32^. Zhang et al.^30^ designed a channel estimation algorithm based on adaptive weighted averaging to suppress noise by averaging the channel coefficients of adjacent OFDM symbols. On this basis, Zhang et al.^31^ proposed a joint sparse channel estimation algorithm based on adaptive average and MSE optimal thresholds for MIMO-OFDM systems, which can be adapted to high-speed mobile scenarios does not require channel prior information. Multi-frame averaging can also be used in SP-based channel estimation. Estrada et al.^15^ estimated the channel after averaging several continuous frames of adjacent OFDM symbols. Muntane and Fernandez^32^ simplified the channel, aiming at the minimum MSE, and obtained the optimal average frame number of OFDM symbols. Multi-frame averaging can suppress the additive noise in the channel effectively, and the superimposed pilot can improve the spectrum efficiency and data rate. Therefore, this paper proposes a weighted averaging channel estimation method based on superimposed pilot, which is named superimposed pilot-weighted averaging (SP-WA). Our main contributions are summarised as follows:

- A new pilot superposition scheme is proposed. Two signals are transmitted, one is superimposed at odd subcarriers, and the other is superimposed at even subcarriers. In this way, data symbols can be transmitted separately and pilot symbols can be contained in all subcarriers.
- An elimination scheme is proposed to remove PDI. The two received signals are subtracted to remove the PDI in preparation for channel estimation.
- A weighted averaging channel estimation method is proposed according to the temporal correlation of wireless channels. On the basis of LS estimation, the channel coefficients of two adjacent OFDM symbols are weighted and averaged to eliminate additive white Gaussian noise (AWGN) and obtain accurate CSI.
- To evaluate the performance of the proposed method, the normalized mean square error (NMSE), BER, and throughput are tested, respectively. Experimental results show that the proposed method has noticeable signal-to-noise ratio (SNR) gains compared with the LS method and the multi-frame averaging (the average number of frames is 2, 4, and 6, respectively) method. The superimposed pilot used in this paper can improve the throughput of the wireless communication system compared with comb pilot.

The remainder of the paper is organized as follows. “System model” section describes the system model. “The proposed method” section introduces the proposed channel estimation and data detection method. Numerical analysis is illustrated in “Numerical results” section. “Conclusion” section concludes the paper.

*Notations* In this paper, *M* and *K* represent the number of OFDM symbols and the total number of subcarriers, respectively; bold italic $\varvec{A}$ represents the matrix; *A*(*k*, *m*) represents the element of the *k*th row and the *m*th column of $\varvec{A}$; $k \in [1,K]$, $m \in [1,M]$; ${k_1}$ and ${k_2}$ are odd and even in the range of [1, *K*], respectively; ${\mathrm{E}}( \cdot )$ represent the mathematical expectation of a random variable; ${V} \sim \fancyscript{C}{{\fancyscript{N}}}(0,{\sigma ^2})$ represent that *V* is a complex Gaussian random variable with zero mean and ${\sigma ^2}$ variance.

## System model

**Figure 1:**
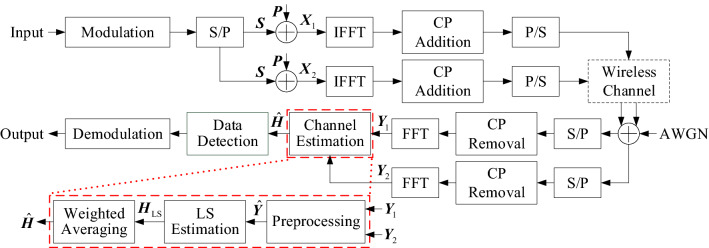
System architecture of the proposed SP-WA method.

It is supposed that one OFDM symbol is transmitted in one frame in this paper. The system architecture of the proposed SP-WA method is shown in Fig. 1.

### Transmitter

**Figure 2:**
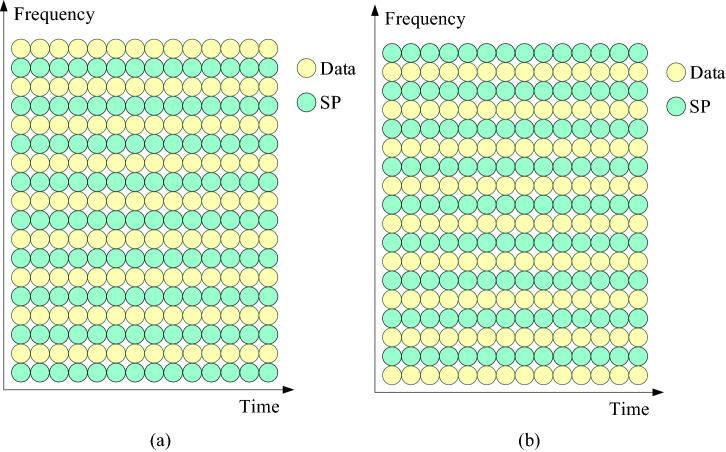
Superimposed pilot pattern in two signals: (**a**) the first signal and (**b**) the second signal.

Figure 1 shows that the input bits are modulated to complex symbols by quadrature phase shift keying (QPSK) or 16-quadrature amplitude modulation (16QAM), and then changed from serial to parallel (S/P), denoted as $\varvec{S}$. $\varvec{S}$ and pilot $\varvec{P}$ are added at different subcarriers to generate two transmitted signals $\varvec{X}_1$ and $\varvec{X}_2$. In Fig. 2, the stacking indexes of $\varvec{X}_1$ and $\varvec{X}_2$ are at the odd subcarriers and the even subcarriers, respectively, that is:

1 $$\begin{aligned} {X_1}({k_1},m)& = {} S({k_1},m) + P({k_1},m) \end{aligned}$$

2 $$\begin{aligned} {X_1}({k_2},m)& = {} S({k_2},m) \end{aligned}$$

3 $$\begin{aligned} {X_2}({k_1},m)& = {} S({k_1},m) \end{aligned}$$

4 $$\begin{aligned} {X_2}({k_2},m)& = {} S({k_2},m) + P({k_2},m) \end{aligned}$$

The *K*-point inverse fast Fourier transform (IFFT) converts $\varvec{X}_1$ and $\varvec{X}_2$ from the frequency domain to the time domain, and subsequently cyclic prefix (CP) is added to avoid ISI before $\varvec{X}_1$ and $\varvec{X}_2$, respectively. Next, $\varvec{X}_1$ and $\varvec{X}_2$ are changed from parallel to serial (P/S). Finally, the two signals are transmitted to a multipath channel with AWGN successively.

### Receiver

It is assumed that the received signal is perfectly synchronized. After S/P and CP removal, the two received signals are transformed from the time domain to the frequency domain by *K*-point fast Fourier transform (FFT), and then $\varvec{Y}_1$ and $\varvec{Y}_2$ are obtained, that is:

5 $$\begin{aligned} {Y_1}({k_1},m)& = {} H({k_1},m)S({k_1},m) + H({k_1},m)P({k_1},m) + {N_1}({k_1},m) \end{aligned}$$

6 $$\begin{aligned} {Y_1}({k_2},m)& = {} H({k_2},m)S({k_2},m) + {N_1}({k_2},m) \end{aligned}$$

7 $$\begin{aligned} {Y_2}({k_1},m)& = {} H({k_1},m)S({k_1},m) + {N_1}({k_1},m) \end{aligned}$$

8 $$\begin{aligned} {Y_2}({k_2},m)& = {} H({k_2},m)S({k_2},m) + H({k_2},m)P({k_2},m) + {N_1}({k_2},m) \end{aligned}$$

where ${N_1}(k,m) \sim {{\fancyscript{C}}}{{\fancyscript{N}}}(0,{\sigma ^2})$. *H*(*k*, *m*) and ${N_1}(k,m)$ denote the channel coefficient and AWGN in the channel, respectively.

## The proposed method

### Channel estimation

As shown in Fig. 1, the two received signals are preprocessed at first. (5) and (8) show that the odd subcarriers of $\varvec{Y}_1$ and the even subcarriers of $\varvec{Y}_2$ have both pilot and data symbols, while (6) and (7) show that the even subcarriers of $\varvec{Y}_1$ and the odd subcarriers of $\varvec{Y}_2$ only have data symbols. Therefore, PDI can be eliminated by the subtraction between $\varvec{Y}_1$ and $\varvec{Y}_2$. That is:

9 $$\begin{aligned} {\tilde{Y}}({k_1},m)& = {} {Y_1}({k_1},m) - {Y_2}({k_1},m) = H({k_1},m)P({k_1},m) + {N_2}({k_1},m) \end{aligned}$$

10 $$\begin{aligned} {\tilde{Y}}({k_2},m)& = {} {Y_1}({k_2},m) - {Y_2}({k_2},m) = - H({k_2},m)P({k_2},m) + {N_2}({k_2},m) \end{aligned}$$

where ${\tilde{Y}}(k,m)$ represents the received signal after preprocessing, ${N_2}(k,m)$ denotes the AWGN in ${\tilde{Y}}(k,m)$, and ${N_2}(k,m) \sim {{\fancyscript{C}}}{{\fancyscript{N}}}(0,2{\sigma ^2})$, respectively. (9) and (10) show that PDI has been removed, and only the interference caused by AWGN remains in ${\tilde{Y}}(k,m)$. The final processed signal ${\hat{Y}}(k,m)$ is obtained by taking the negative values on the even subcarriers:

11 $$\begin{aligned} {\hat{Y}}({k_1},m)& = {} {\tilde{Y}}({k_1},m) \end{aligned}$$

12 $$\begin{aligned} {\hat{Y}}({k_2},m)& = {} - {\tilde{Y}}({k_2},m) \end{aligned}$$

The coarse channel frequency response (CFR) is obtained by LS estimation:

13 $$\begin{aligned} {H_{{\mathrm{LS}}}}(k,m) = \frac{{{\hat{Y}}(k,m)}}{{P(k,m)}} = H(k,m) + {N_{{\mathrm{LS}}}}(k,m) \end{aligned}$$

where ${N_{{\mathrm{LS}}}}(k,m)$ represents the estimated error of CFR.

AWGN in the channel has always been a key factor affecting the accuracy of channel estimation^33^, and a channel estimation method to deal with AWGN has been proposed in^34^. Multi-frame averaging is a method to deal with AWGN too. In quasi-static channels, CFR changes slowly with time, so it can be considered that CFR does not change within *F* OFDM symbols. Therefore, the average of the adjacent *F* channel coefficients will only reduce the noise power. Based on LS estimation, the average of multi-frame can be expressed as:

14 $$\begin{aligned} {H_{\mathrm{A}}}(k,m) = \frac{1}{F}\sum \limits _{i = m - F + 1}^m {{H_{{\mathrm{LS}}}}(k,i)} \end{aligned}$$

Substituting (13) into (14), we have:

15 $$\begin{aligned} {H_{\mathrm{A}}}(k,m) = \frac{1}{F}\sum \limits _{i = m - F + 1}^m {H(k,i) + {N_{{\mathrm{LS}}}}(k,i)} = H(k,m) + \frac{1}{F}\sum \limits _{i = m - F + 1}^m {{N_{{\mathrm{LS}}}}(k,i)} \end{aligned}$$

(15) shows that, at the *m*th OFDM symbol, the channel coefficient does not change, and the power of AWGN is decreased by a factor of *F*, which reduces the interference of AWGN. The more average frames are selected, the higher the channel estimation accuracy will be, and the more data the computer needs to cache. To reduce the amount of data cached by the computer and improve the accuracy of channel estimation, a weighted scheme based on the average of two frames is introduced and a weighting factor by the correlation between the *m*th and $(m - 1)$th OFDM symbols is set in this paper. The final channel estimation result can be expressed as:

16 $$\begin{aligned} {\hat{H}}(k,m) = \left\{ \begin{array}{l} {H_{{\mathrm{LS}}}}(k,m),\\ \alpha {H_{{\mathrm{LS}}}}(k,m) + (1 - \alpha ){\hat{H}}(k,m - 1), \end{array} \right. \begin{array}{*{20}{c}} {m = 1}\\ {m > 1} \end{array} \end{aligned}$$

where $\alpha$ is the weighting factor. The method can improve the channel estimation accuracy effectively and prepare for data detection in “Data detection” section. Taking NMSE and BER as evaluation criteria, the specific experimental results of channel estimation accuracy are given in “Analysis of channel estimation accuracy” section.

### Data detection

According to (6) and (7), there are only data symbols at the indices of even subcarriers of $\varvec{Y}_1$ and odd subcarriers of $\varvec{Y}_2$, hence $\varvec{Y}_2$ and $\varvec{Y}_1$ are used to recover data symbols at odd and even subcarriers, respectively. The LS equalizer is used to obtain the data:

17 $$\begin{aligned} {\hat{S}}({k_1},m)& = {} \frac{{{Y_2}({k_1},m)}}{{{\hat{H}}({k_1},m)}} \end{aligned}$$

18 $$\begin{aligned} {\hat{S}}({k_2},m)& = {} \frac{{{Y_1}({k_2},m)}}{{{\hat{H}}({k_2},m)}} \end{aligned}$$

The output bits are obtained by QPSK or 16QAM demodulation of ${\hat{S}}(k,m)$, and the BER of the proposed method can be obtained by comparing the output bits with the input bits.

## Numerical results

We consider a single input single output-OFDM (SISO-OFDM) system with $K = 1024$ subcarriers. The quasi-static channel is assumed, and the parameters used in this section are summarized in Table 1. We perform tests in China digital television test 1st (CDT 1) and CDT 6^35^ channels, respectively. These channels are all Rayleigh channels with the taps number of 6, whose power delay profiles are shown in Table 2.

**Table 1:** Simulation parameters of the OFDM system.

Parameters	Specifications
System model	SISO-OFDM
Channel distribution	Rayleigh
Baseband symbol rate	7.56 MHz
Modulation mode	QPSK/16QAM
Taps number	6
OFDM frames number	100
Subcarriers number	1024
CP length (subcarriers)	256

**Table 2:** Power delay profile for CDT 1 and CDT 6 multipath fading channels.

Tap	CDT 1		CDT 6	
	Delay ($\mu$s)	Power (dB)	Delay ($\mu$s)	Power (dB)
1	$- 1.8$	$- 20$	$- 18$	$- 10$
2	0	0	$- 1.8$	$- 20$
3	0.15	$- 20$	0	0
4	1.8	$- 10$	0.15	$- 20$
5	5.7	$- 14$	1.8	$- 10$
6	18	$- 18$	5.7	$- 14$

We first give the procedure of determining the weighting factor $\alpha$. Then, numerical results are given in terms of NMSE and BER, respectively, to prove that the proposed method can improve the channel estimation accuracy. NMSE is expressed as:

19 $$\begin{aligned} {e_{{\mathrm{NMSE}}}} = \sqrt{\frac{{{\mathrm{E}}(|\varvec{h} - \hat{\varvec{h}}{|^2})}}{{{\mathrm{E}}(|\varvec{h}{|^2})}}} \end{aligned}$$

where $\varvec{h}$ and $\varvec{{\hat{h}}}$ represent the real channel impulse response (CIR) and the CIR obtained by various channel estimation methods, respectively. BER is expressed as:

20 $$\begin{aligned} {r_{{\mathrm{BER}}}} = \frac{n_{\mathrm{E}}}{n_{\mathrm{T}}} \end{aligned}$$

where ${n_{\mathrm{E}}}$ and ${n_{\mathrm{T}}}$ represent the number of error bits and the number of transmitted bits, respectively. Moreover, we also analyze the impact of the superimposed pilot on the throughput of the system, which can be expressed as^15^:

21 $$\begin{aligned} {{R_{\mathrm{Throup}}} = \frac{K - {N_{\mathrm{P}}}}{K} \cdot \frac{{{N_{\mathrm{B}}}(1 - {r_{\mathrm{BER}}})}}{T}} \end{aligned}$$

where, ${N_{\mathrm{P}}}$ represents the number of pilot subcarriers, ${N_{\mathrm{B}}}$ represents the number of transmitted bits per OFDM symbol, and *T* is the transmission time and assumed as $T=1$.

### Determination of $\alpha$

**Figure 3:**
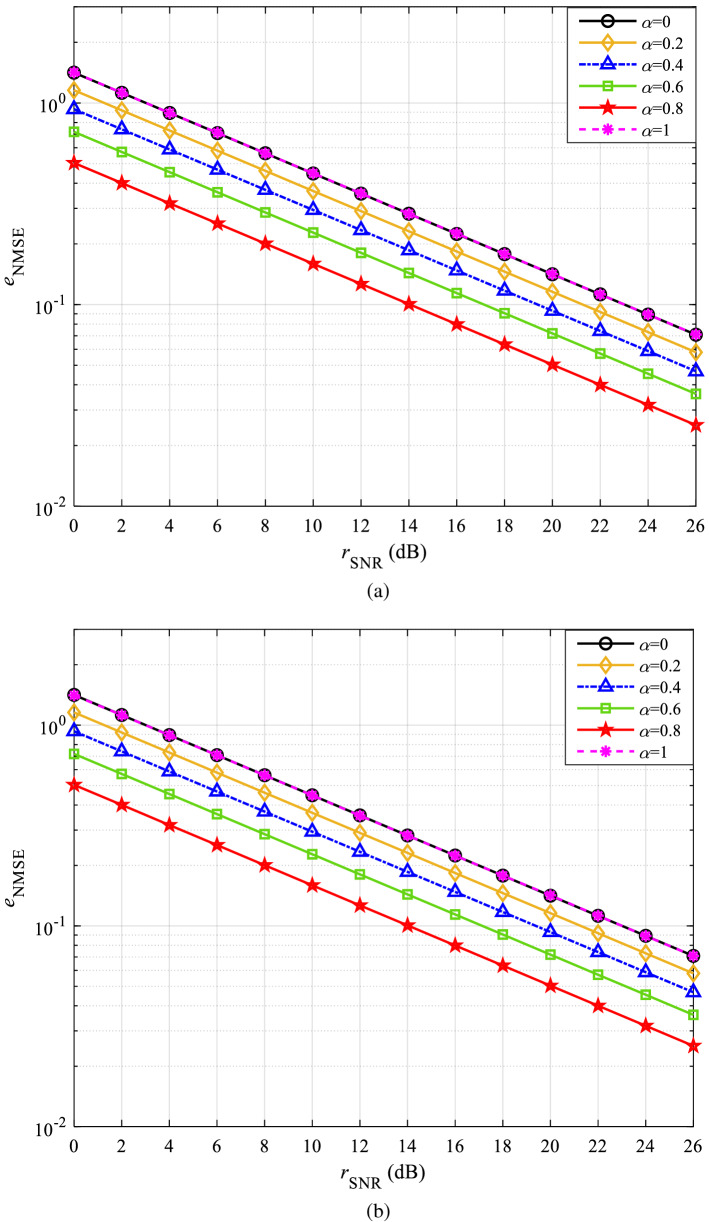
NMSE curves with $\alpha$ within 0 to 1: **(a**) CDT 1 and (**b**) CDT 6.

According to (16), the maximum value of $\alpha$ is 1 and the minimum value is 0. When $\alpha =1$, the final estimation of the channel is the LS estimation of the channel. When $\alpha = 0$, the signal experiences all of the same changes in the channel. Obviously, when $\alpha = 1$ or $\alpha = 0$, the error of channel estimation is maximum. With an interval of 0.2, the obtained NMSE simulation curves of $\alpha$ within 0 to 1 in CDT 1 and CDT 6 channels are shown in Fig. 3a,b, respectively. Since the proposed method is applicable to a variety of wireless channels, the trend of the final simulation is similar. Therefore, the simulation results of CDT 1 channel are taken as an example to elaborate.

**Figure 4:**
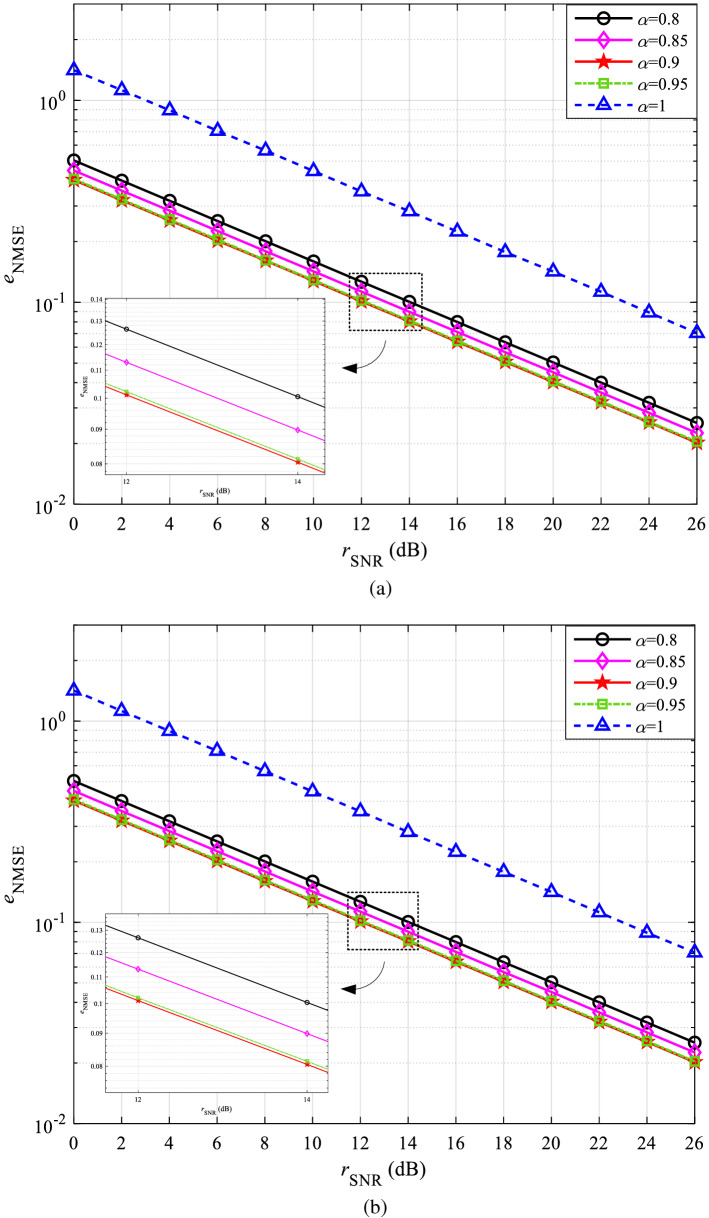
NMSE curves with $\alpha$ within 0.8 to 1: (**a**) CDT 1 and (**b**) CDT 6.

Figure 3a shows that the curves of $\alpha = 1$ and $\alpha = 0$ overlap and both have the worst performance, which also validates the previous inference. With the increase of $\alpha$, the estimation of CSI is more accurate, and there is a minimum value of ${e_{{\mathrm{NMSE}}}}$ when $\alpha$ is between 0.8 and 1. For this purpose, the NMSE simulation curves of $\alpha$ within 0.8 to 1 in CDT 1 and CDT 6 channels obtained with an interval of 0.05 are shown in Fig. 4a,b, respectively. The simulation result of the CDT 1 channel is still illustrated in detail as an example.

In Fig. 4a, the curves at $\alpha =0.9$ and $\alpha =0.95$ are close to overlap. Local magnification shows that ${e_{{\mathrm{NMSE}}}}$ is smaller at $\alpha =0.9$. When the value of $\alpha$ is close to 0.9, the slight change in the value of $\alpha$ has no obvious influence on the accuracy of channel estimation, so the weighting factor $\alpha$ of the experiment is finally selected as 0.9.

### Analysis of channel estimation accuracy

At the transmitter, the comb pilot and SP schemes are adopted, respectively. At the receiver, LS method, multi-frame averaging method, and weighted averaging method are adopted to estimate the channel, respectively. In this paper, the proposed method is tested in quasi-static channels. To fully compare the channel estimation accuracy of different methods, the frame numbers of the multi-frame averaging method are taken as 2, 4, 6, and 100, respectively. Seven simulation curves are obtained, which are named Comb-LS, SP-LS, SP-2FA, SP-4FA, SP-6FA, SP-100FA, and SP-WA, respectively.

#### NMSE

**Figure 5:**
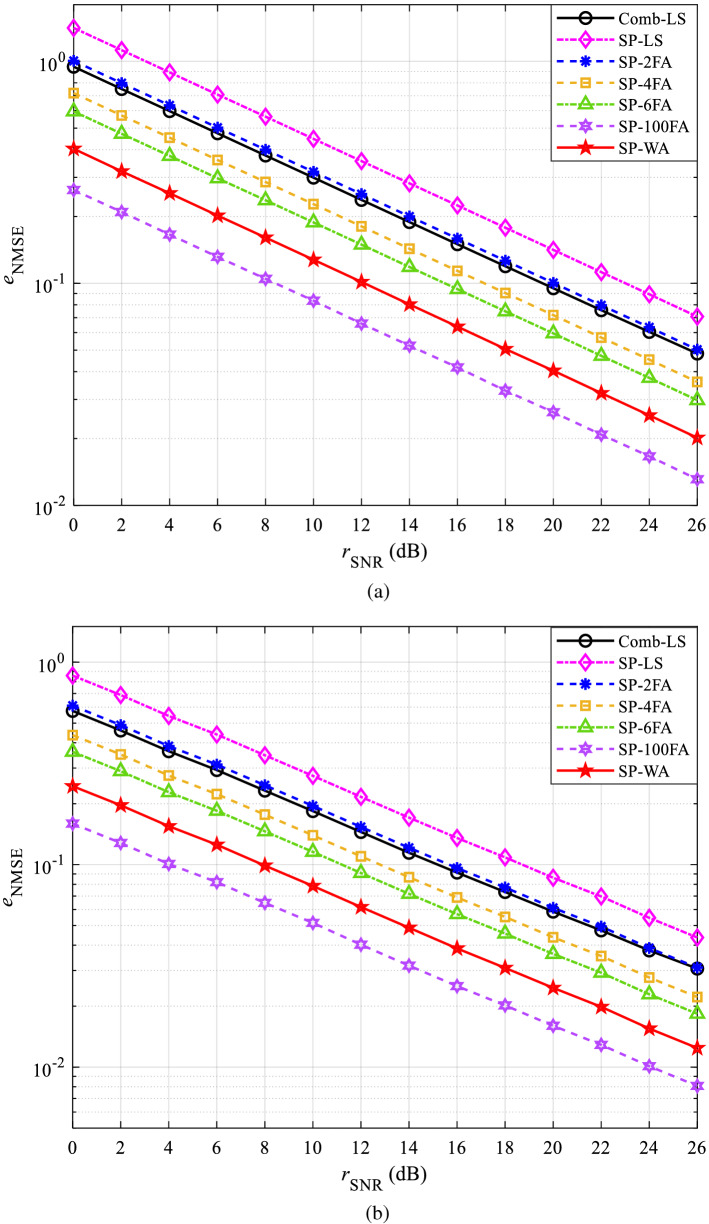
The NMSE performance in CDT 1 channel: (**a**) QPSK and (**b**) 16QAM.

**Figure 6:**
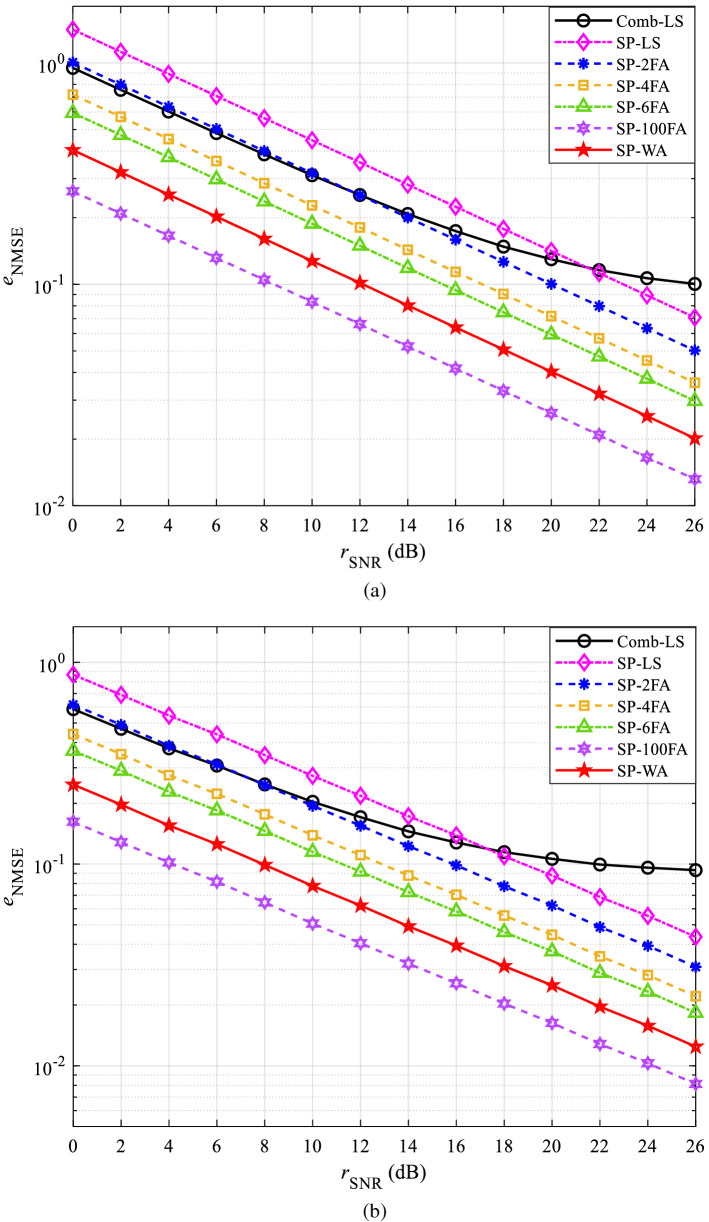
The NMSE performance in CDT 6 channel: (**a**) QPSK and (**b**) 16QAM.

The performance curves of NMSE in CDT 1 and CDT 6 channels are shown in Figs. 5 and 6, respectively. Figures 5a and 6a are obtained under QPSK modulation, Figs. 5b and 6b are obtained under 16QAM modulation.

Figure 5a shows that the NMSE of all methods decreases with the increase of SNR, and the performance of SP-LS is the worst. This is because the power of AWGN has doubled after the data are preprocessed at the receiver. In the multi-frame averaging channel estimation, SP-100FA has better channel estimation accuracy than SP-6FA, SP-4FA, and SP-2FA, this is because the power of AWGN decreases with the increase of the average frame number. The reason that the channel estimation accuracy of the proposed SP-WA is better than that of SP-6FA, SP-4FA, and SP-2FA is that SP-WA considers the weights between two adjacent OFDM symbols instead of simply averaging. The reason why the channel estimation accuracy of SP-WA is inferior to that of SP-100FA is that SP-100FA averages all OFDM symbols. However, SP-100FA needs to cache a lot of data, whereas SP-WA does not. Figure 5b shows that the downward trend of the simulation curve obtained with 16QAM modulation is the same as that obtained with QPSK modulation, but the ${e_{{\mathrm{NMSE}}}}$ is smaller.

Similar results can be obtained in Fig. 6a,b, but the Comb-LS flattens out when SNR is large, which is determined by the characteristics of the CDT 6 channel. However, SP scheme weakens the inherent influence of channel on signal when preprocessing at the receiver, so this phenomenon does not appear in the proposed method.

#### BER

**Figure 7:**
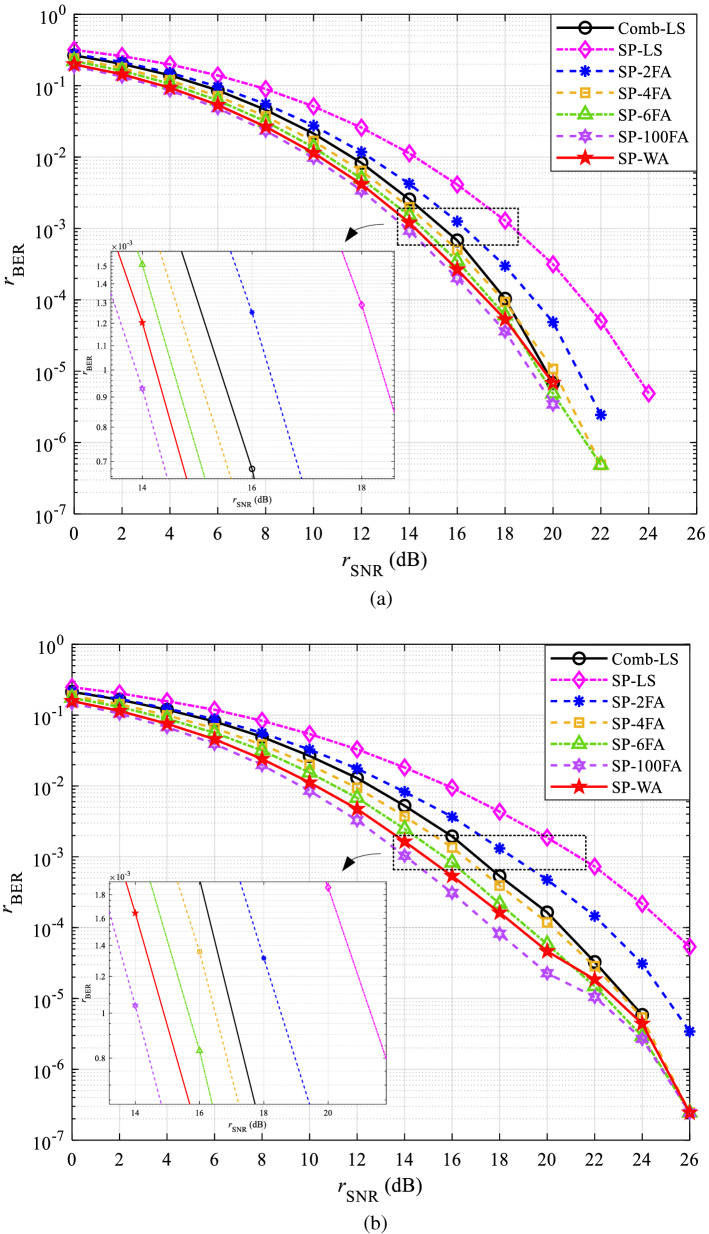
The BER performance in CDT 1 channel: (**a**) QPSK and (**b**) 16QAM.

**Figure 8:**
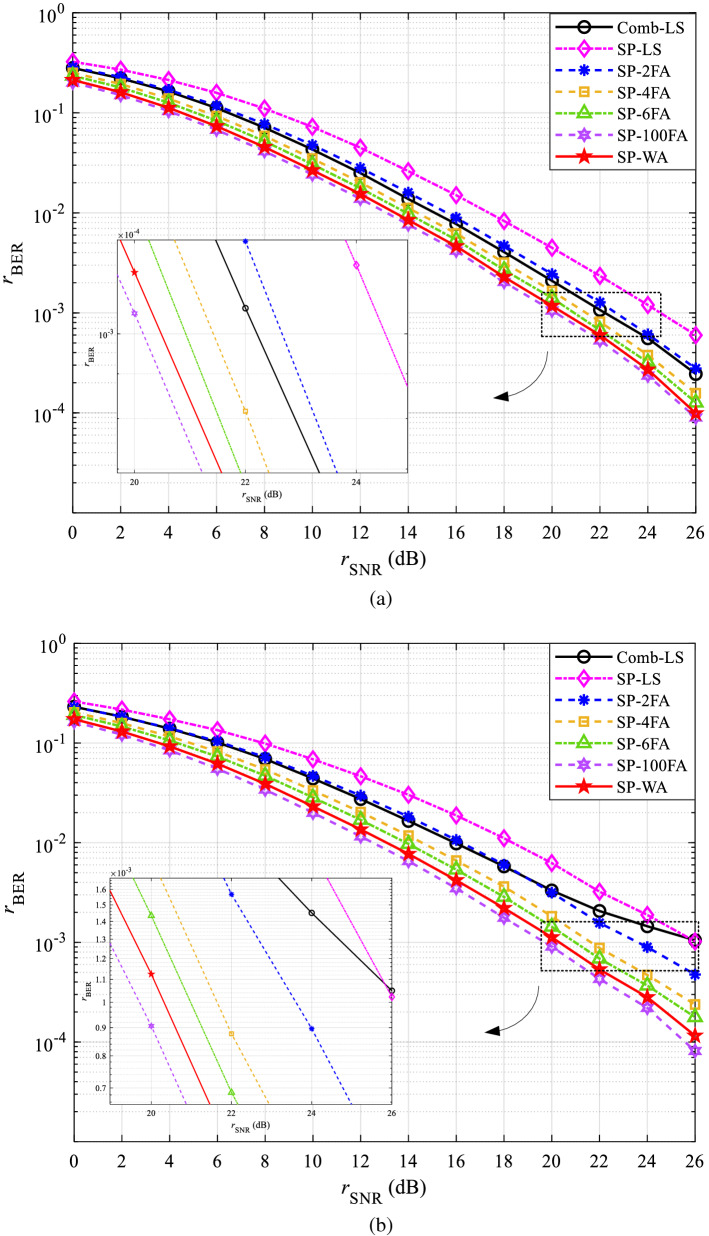
The BER performance in CDT 6 channel: (**a**) QPSK and (**b**) 16QAM.

The performance curves of BER in CDT 1 and CDT 6 channels are shown in Figs. 7 and 8, respectively. Figures 7a and 8a are obtained under QPSK modulation, Figs. 7b and 8b are obtained under 16QAM modulation.

In Fig. 7a, the BER of all methods shows a decrease with the increase of SNR. This is because with the increase of SNR, the interference of AWGN in the channel on the signal will be reduced, so the accuracy of channel estimation will be higher. Similar experimental results are also presented in Fig. 7b, which also proves that the proposed method is applicable to different modulation modes from the perspective of BER.

Figure 8a compared with Fig. 7a, Fig. 8b compared with Fig. 7b, the accuracy of channel estimation is obviously decreased, which is caused by the selectivity of the CDT 6 channel. To further demonstrate the effectiveness of the proposed method in BER, the specific SNR gains of SP-WA than that of other channel estimation methods at the BER of ${10^{ - 3}}$ are shown in Table 3.

**Table 3:** The SNR gains of SP-WA than that of other methods at the BER of ${10^{ - 3}}$ (dB).

Methods	CDT1		CDT6	
	QPSK	16QAM	QPSK	16QAM
SP-100FA	− 0.26	− 0.82	− 0.35	− 0.56
SP-6FA	0.30	0.72	0.40	0.82
SP-4FA	0.74	1.60	0.88	1.61
Comb-LS	1.11	2.17	1.61	5.58
SP-2FA	2.06	3.67	2.07	3.20
SP-LS	4.11	6.24	3.89	5.31

Table 3 shows that, compared with SP-100FA, the SNR gains of SP-WA is less than 0, indicating that the channel estimation accuracy of SP-WA is worse, but SP-100FA does not improve much compared with SP-WA. Moreover, SP-WA does not need to cache a large amount of data, which is beneficial to practical applications. Except for the first row, the other values in Table 3 are positive, indicating that SP-WA has higher channel estimation accuracy than SP-6FA, SP-4FA, Comb-LS, SP-2FA, and SP-LS, respectively. Comparing the SNR gains of different modulation mode in the same channel scenario, it can be seen that the proposed method is more suitable for high-order modulation.

### Analysis of throughput

**Figure 9:**
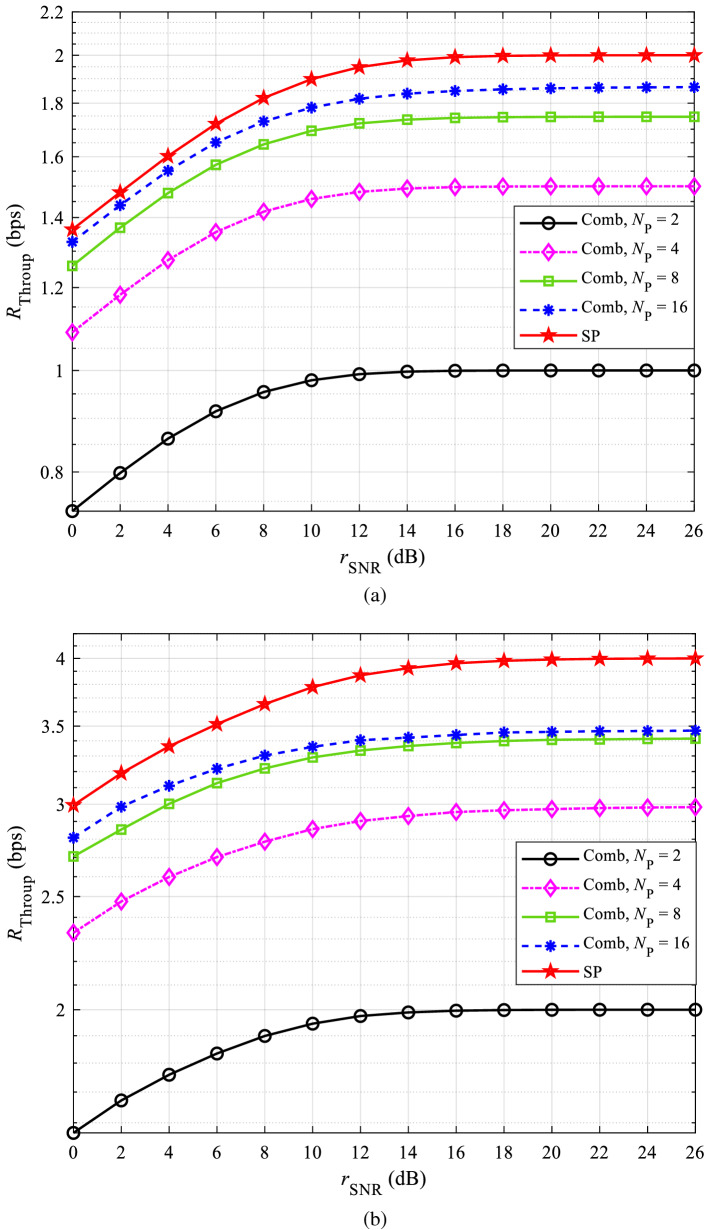
Throughput comparison of SP and comb pilot with different pilot intervals: (**a**) QPSK and (**b**) 16QAM.

To clarify that SP can improve spectral efficiency than comb pilot, the throughput is analyzed in this subsection. Figure 9 shows the throughput comparison between SP and comb pilot, in which Fig. 9a is under the QPSK modulation mode, and Fig. 9b is under the 16QAM modulation mode.

Figure 9a shows that, the larger the comb pilot interval is, the higher the throughput will be. The throughput of comb pilot will approach the throughput of SP with the gradual increase of comb pilot interval, but will not reach the throughput that SP can bring. The reason is that, with superimposed pilot, $[(K - {N_{\mathrm{P}}})/K]$ is equal to 1, whereas with comb pilot, $[(K - {N_{\mathrm{P}}})/K]$ can approach to 1 but not equal to 1. The same behavior is obtained in Fig. 9b. Comparing Fig. 9a,b, it can be concluded that higher throughput can be obtained in 16QAM than in QPSK.

## Conclusion

To improve the spectrum efficiency and the channel estimation accuracy of wireless communication systems, a weighted averaging channel estimation method based on superimposed pilot is proposed. Firstly, the superposition of data and pilot is adopted to improve the spectrum efficiency of the communication system. Then, the PDI is eliminated by the subtraction between received signals. Finally, the weighted averaging method is performed to improve the performance of removing AWGN.

Simulation results demonstrate that the proposed SP-WA method can significantly improve the channel estimation accuracy than SP-6FA, SP-4FA, Comb-LS, SP-2FA, and SP-LS, respectively. Although the channel estimation accuracy of SP-WA is inferior to that of SP-100FA, it does not need to cache a large amount of data for averaging. Therefore, the proposed SP-WA is more beneficial to practical applications. Compared with comb pilot, the superimposed pilot used in this paper can improve the throughput of the system and save frequency band resources. SP-WA can be used in different channel scenarios, and is more suitable for high-order modulation, which provides convenience for improving data transmission rate.
